# FOXP3 Activates SUMO-Conjugating *UBC9* Gene in MCF7 Breast Cancer Cells

**DOI:** 10.3390/ijms19072036

**Published:** 2018-07-13

**Authors:** Chiung-Min Wang, William H. Yang, Runhua Liu, Lizhong Wang, Wei-Hsiung Yang

**Affiliations:** 1 Department of Biomedical Sciences, Mercer University School of Medicine, Savannah, GA 31404, USA; meowy200@yahoo.com (C.-M.W.); meowy100@yahoo.com (W.H.Y.); 2 Department of Genetics and Comprehensive Cancer Center, University of Alabama at Birmingham, Birmingham, AL 35294, USA; runhua@uab.edu (R.L.); lwang12@uab.edu (L.W.)

**Keywords:** FOXP3, UBC9, transcriptional activity, phosphorylation, acetylation, ubiquitination, SUMOylation

## Abstract

Forkhead Box Protein P3 (FOXP3), a transcription factor of the FOX protein family, is essentially involved in the development of regulatory T (Treg) cells, and functions as a tumor suppressor. Although FOXP3 has been widely studied in immune system and cancer development, its function in the regulation of the *UBC9* gene (for the sole E2 enzyme of SUMOylation) is unknown. Herein, we find that the overexpression of FOXP3 in human MCF7 breast cancer cells increases the level of *UBC9* mRNA. Moreover, the level of UBC9 protein dose-dependently increases in the FOXP3-Tet-off MCF7 cells. Notably, the promoter activity of the *UBC9* is activated by FOXP3 in a dose-dependent manner in both the MCF7 and HEK293 cells. Next, by mapping the *UBC9* promoter as well as the site-directed mutagenesis and ChIP analysis, we show that the FOXP3 response element at the −310 bp region, but not the −2182 bp region, is mainly required for *UBC9* activation by FOXP3. Finally, we demonstrate that the removal of phosphorylation (S418A and Y342F) and the removal of acetylation/ubiquitination (K263R and K263RK268R) of the FOXP3 result in attenuated transcriptional activity of *UBC9*. Taken together, FOXP3 acts as a novel transcriptional activator of the human *UBC9* gene, suggesting that FOXP3 may have physiological functions as a novel player in global SUMOylation, as well as other post-translational modification systems.

## 1. Introduction

Transcription factor Forkhead Box Protein P3 (FOXP3), encoded from an X chromosome, was originally identified as the causative mutation for lethal X-linked autoimmunity-allergic dysregulation syndrome [1,2,3,4]. In the T cell lineage, FOXP3 is required for regulatory T (Treg) cell development and the maintenance of immune homeostasis [5]. Therefore, FOXP3 has gradually become the most specific biomarker of Treg cells in the immunosuppressive system. The animal model studies have suggested that truncated and/or deficient FOXP3 is lethal, as a result of Treg cell deficiency [6,7]. In humans, mutations of the *FOXP3* gene are associated with a rare autoimmune disorder termed immunodysregulation, poly-endocrinopathy and enteropathy, X-linked syndrome (IPEX) [4].

Previous studies have shown that FOXP3 also expresses in a variety of normal tissues, such as breast, lung, prostate, and thymus [8,9,10], suggesting that FOXP3 might have broad biological functions. Recent extensive studies have strongly suggested that FOXP3 is a novel tumor suppressor in breast and prostate cancers. For example, a germline mutation of FOXP3 results in a high rate of spontaneous breast cancer in mice [11]. In addition, deletions and mutations of FOXP3 have been found in human breast cancer samples. Secondly, FOXP3 represses several key target genes in cancer development, such as *BRCA1* [12], *CD44* [13], *HER2/ERBB2* [11], and *SKP2* [14], providing a strong link between FOXP3 and DNA repair system, as well as FOXP3 and cell cycle regulation. Thirdly, the FOXP3-miR-146-NF-κB axis has a functional role during tumor initiation in both breast and prostate cancers [15,16]. Moreover, a transcriptional axis of FOXP3-BRCA1-miR-155 in breast cancer cells has been reported, suggesting that plasma miR-155 may serve as a non-invasive biomarker for the detection of early stage breast cancer [17]. Finally, miR-141 and miR-200c are regulated by a FOXP3-KAT2B axis in breast cancer cells, suggesting that circulating levels of miR-141 and miR-200c are potential biomarkers for the early detection of breast cancer metastases [18]. Overall, these results demonstrate that FOXP3 is a novel X-linked tumor suppressor and transcriptional repressor for breast and prostate cancers, suggesting a unique therapeutic opportunity for cancer treatment.

A previous study demonstrates that the deletion of UBC9 (the only E2 enzyme for SUMOylation) in Treg cells leads to early-onset lethal autoimmune diseases, suggesting that protein SUMOylation is required for regulatory T cell expansion and function [19]. Moreover, UBC9 is required for the late-stage maturation of thymocytes [20]. Although FOXP3 has an essential and critical role in Treg development, autoimmunity, and cancer development, and hundreds of FOXP3 target genes have been identified in both Treg cells and cancer cells, the functional role of FOXP3 in regulating post-translational modification system is still largely unknown. As FOXP3 is a transcription factor and SUMOylation has profound effects on regulating normal cell physiology, development, and tumorigenesis [21,22,23,24,25,26,27,28], we assessed the function of FOXP3 in *UBC9* transcriptional activity using MCF7 human breast cancer cells in the present study.

## 2. Results

### 2.1. FOXP3 Increases UBC9 mRNA and Protein Levels

**Figure 1:**
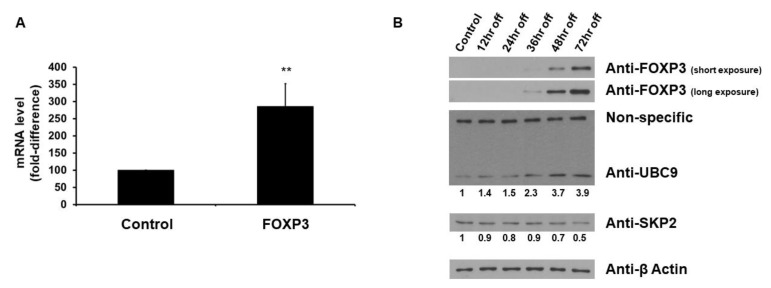
Forkhead Box Protein P3 (FOXP3) increases the UBC9 level. (**A**) Real-time RT-PCR analysis of *UBC9* expression by FOXP3 from MCF7 human breast cancer cells. Total RNA was extracted and reverse transcribed to cDNA, followed by qPCR analysis with glyceraldehyde-3-phosphate dehydrogenase (GAPDH) as an internal control. Each point represents the average of three experiments, each with triplicate samples. Error bars indicate standard errors. ** indicates *p* < 0.001; (**B**) Western blot analysis of UBC9 expression from FOXP3-Tet-off MCF7 cells. The expression levels of FOXP3, UBC9, and SKP2 in FOXP3-Tet-off MCF7 cells were determined using anti-FOXP3, anti-UBC9, and anti-SKP2 immunoblotting, respectively. The β-Actin levels were also determined for equal loading.

To determine whether FOXP3 affects the levels of *UBC9* mRNA in MCF7 human breast cancer cells, expression vectors encoding wild-type *FOXP3* or empty vectors were transfected into MCF7 cells. As shown in Figure 1A, when the wild-type *FOXP3* was transfected, the level of *UBC9* mRNA was increased (approximately 3-fold). We next used FOXP3-Tet-off MCF7 cells to evaluate whether FOXP3 affects the UBC9 protein expression. As shown in Figure 1B, the FOXP3 induction by doxycycline removal increased the expression levels of UBC9, while the FOXP3 induction decreased the level of SKP2 (consistent with the previous report that FOXP3 represses SKP2 expression [14]). These results indicate that FOXP3 enhances the UBC9 expression in MCF7 human breast cancer cells.

### 2.2. FOXP3 Is an Activator of the UBC9 Promoter

**Figure 2:**
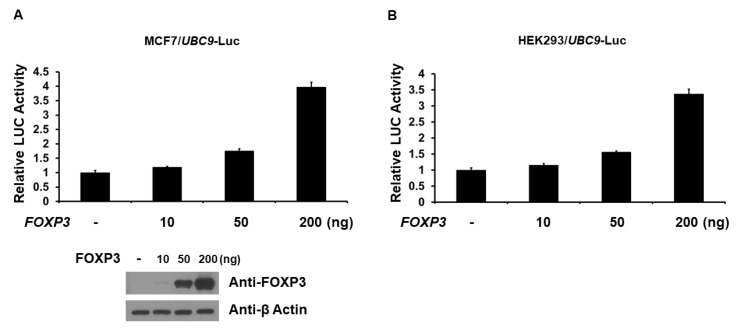
FOXP3 activates *UBC9* transcription. MCF7 (**A**) and HEK293 (**B**) cells were co-transfected with *UBC9* luciferase plasmid and a different amount of *FOXP3* expression plasmid. Cells were cultured for 48 h after transfection. Luciferase activities (LUC) were measured and normalized with Renilla activity. Relative LUC activity (presented as fold activation) was calculated and plotted on a graph. The levels of FOXP3 and β-Actin proteins were determined by Western blot analysis. The experiments were performed three times in triplicate setting. Error bars indicate the standard error.

**Figure 3:**
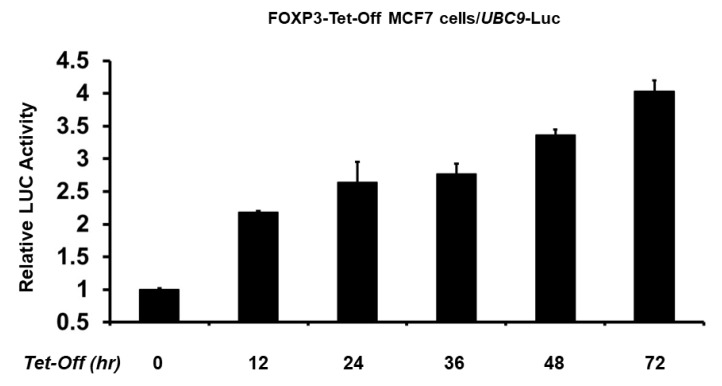
FOXP3 enhances *UBC9* transcription in FOXP3-Tet-off MCF7 cells. FOXP3-Tet-off MCF7 cells were transfected with *UBC9*-firefly luciferase plasmid. Cells were cultured for 48 h after transfection. Luciferase activities (LUC) were measured and normalized with Renilla activity. Relative LUC activity (presented as fold activation) was calculated and plotted on a graph. The experiments were performed three times in triplicate setting. Error bars indicate the standard error.

As FOXP3 increases UBC9 expression in the MCF7 cells, we next determined whether the *UBC9* promoter is regulated by FOXP3. The −2392 bp *UBC9* promoter-firefly luciferase reporter plasmid was cotransfected with wild-type *FOXP3* into MCF7 and HEK293 cells, and the *UBC9* promoter activity was determined by measuring the luciferase (LUC) activity in cell lysates, 48 h after transfection. As shown in Figure 2, FOXP3 dose-dependently activated *UBC9* gene transcription in the luciferase assays using MCF7 and HEK293 cells. In order to confirm the FOXP3-mediated *UBC9* promoter activity, we next measured the *UBC9* promoter activity using FOXP3-Tet-off MCF7 cells. As shown in Figure 3, the FOXP3 induction by doxycycline removal activated the *UBC9* promoter activity in the luciferase assays, using FOXP3-Tet-off MCF7 cells. These findings indicate that FOXP3 is an activator of the *UBC9* transcription.

### 2.3. Minimal UBC9 Promoter Region Responsive to FOXP3 Activation

**Figure 4:**
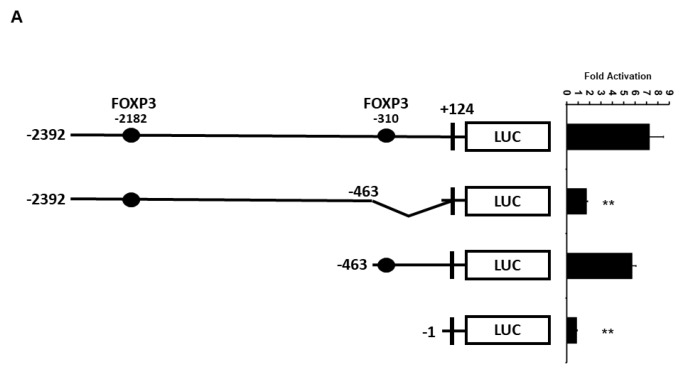

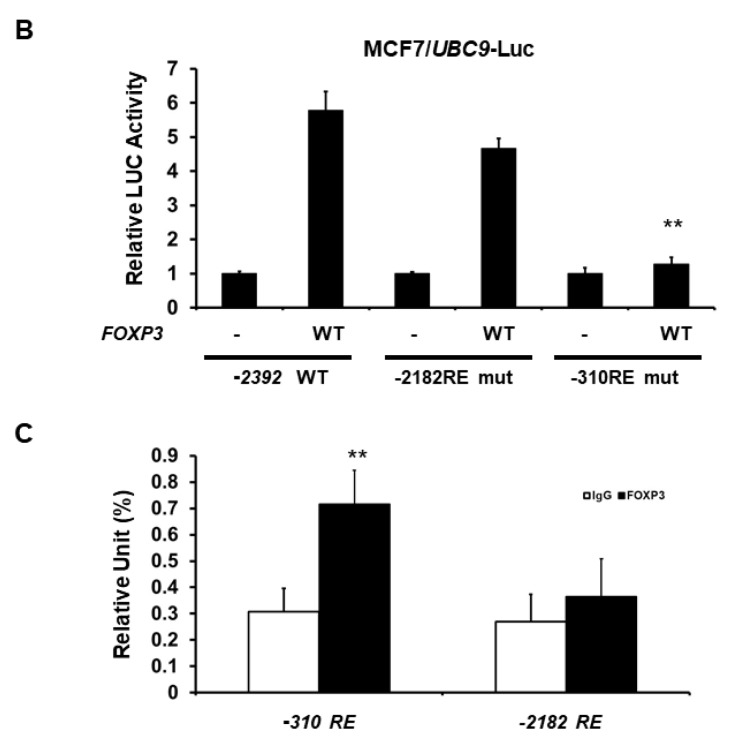
Regions of *UBC9* promoter important for transcriptional activation by FOXP3. (**A**) MCF7 cells were co-transfected with *UBC9* promoter deletion constructs and *FOXP3* expression plasmid. Luciferase activities were measured 48 h after transfection and normalized with Renilla activity. Relative LUC activity (fold activation) was calculated and plotted. The experiments were conducted three times and in triplicate. Error bars indicate the standard error. ** indicates *p* < 0.001 vs. −2392 bp construct; (**B**) MCF7 cells were co-transfected FOXP3 plasmid and with either −2392 wild-type (WT), −2182 RE mutated, or −310 RE mutated *UBC9* promoter constructs. Luciferase activities (LUC) were measured and normalized with Renilla activities after transfection. Relative LUC activity (fold activation) was calculated and plotted. The experiments were conducted three times and in triplicate. Error bars indicate the standard error. ** indicates *p* < 0.001 vs. −2392 bp WT construct; (**C**) quantification of the amount of DNA precipitated (expressed as a percentage of the total input DNA) in ChIP analysis of FOXP3-binding sites in the *UBC9* promoter region in the MCF7 cells. The experiments were performed three times. ** indicates *p* < 0.001 vs. IgG.

Because we found that the −2392 bp *UBC9* promoter contains two FOX response elements (around the −310 and −2182 bp regions), the *UBC9* promoter was truncated to determine the minimal region that is essential for the transcriptional activation by FOXP3. As shown in Figure 4A, two promoters of different lengths (−2392 and −463 bp) showed similar transcriptional activity by FOXP3. However, the deletion of the promoter region (from −463 to +124 bp), which contains the proximal FOX response element, resulted in a significant loss (approximately 75% loss) of the FOXP3-mediated transcriptional activity in the MCF7 cells.

To further investigate whether the −310 bp region is required for FOXP3-mediated activation, we next generated −310 bp mutant (GCCAACA → GCCGGCA) and −2182 bp mutant (GTTGGC → GCCGGC) *UBC9* promoter-LUC plasmids. Consistent with the result in Figure 4A, mutation of the −310 bp response element (RE) (but not mutation of −2182 bp region) dramatically reduced the FOXP3-mediated *UBC9* promoter activity (Figure 4B). To confirm that FOXP3 directly binds to the UBC9 promoter region, we next performed immunoglobin (IgG) or FOXP3 chromatin immunoprecipitation (ChIP) assay with qPCR analysis. As shown in Figure 4C, FOXP3 mainly binds to the −310 bp response element. This result indicates that the proximal FOXP3 response element (approximately −310 bp region) is the major FOXP3 binding site and is more important for the FOXP3 action on the *UBC9* promoter.

### 2.4. Post-Translational Modifications of FOXP3 Are Involved for FOXP3-Mediated UBC9 Transcriptional Activity

**Figure 5:**
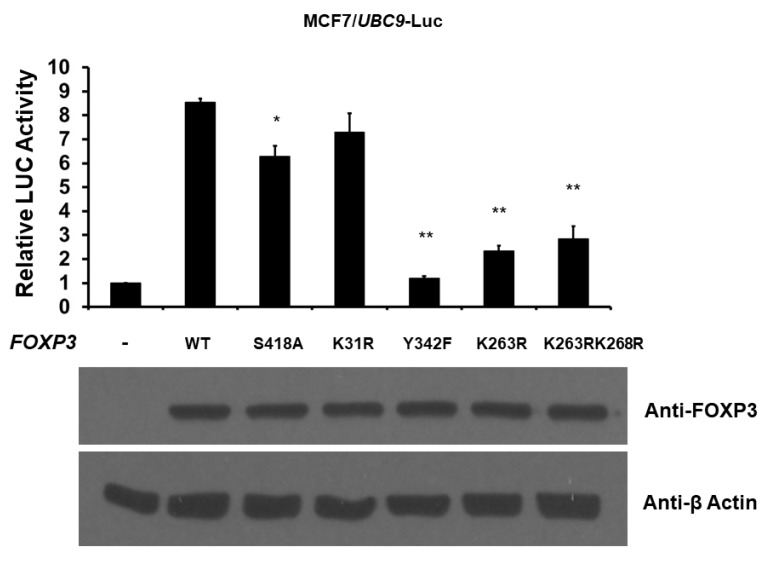
Post-translational modifications of FOXP3 regulate the activation of the *UBC9* promoter. MCF7 cells were co-transfected with a reporter plasmid with the *UBC9* promoter and either wild-type (WT), S418A, K31R, Y342F, K263R, or K263RK268R *FOXP3* expression plasmid. The luciferase activities (LUC) were measured and normalized with Renilla activity, 48 h after transfection. The relative LUC activity (presented as fold activation) was calculated and plotted on a graph. The protein levels of FOXP3 in the MCF7 cells from the reporter assays were confirmed using anti-FOXP3 immunoblotting. The level of β-Actin protein serves as a loading control. The reporter assays were performed three times with similar results. * indicates *p* < 0.05 vs. WT. ** indicates *p* < 0.001 vs. WT.

Because FOXP3 has been shown to be phosphorylated at S418 [29] and Y342 [30], acetylated at K31 [31], and acetylated/ubiquitinated at K263 and K268 [31], we next examined the effect of the post-translational modifications of FOXP3 on the transcriptional activity of the *UBC9* promoter. We cotransfected the *UBC9*-firefly luciferase reporter plasmid with either wild-type, S418A (mimicking de-phosphorylated), Y342F (mimicking de-phosphorylated), K31R (mimicking de-acetylated), K263R (mimicking de-acetylated/ubiquitinated), or K263RK268R (mimicking de-acetylated/ubiquitinated) *FOXP3* expression plasmid, and determined luciferase activity 48 h post-transfection. As shown in Figure 5, while the wild-type and K31R FOXP3 enhanced 7–8-fold *UBC9* promoter activity, S418A, Y342F, K263R, and K263RK268R FOXP3 significantly reduced this effect (approximately 27% loss for S418A, 86% loss for Y342F, 73% loss for K263R, and 67% loss for K263RK268R). This result suggests that the phosphorylation, acetylation, and ubiquitination of FOXP3 are involved for the FOXP3-mediated *UBC9* promoter activity.

## 3. Discussion

Transcription factors regulate the downstream target genes by responding to a wide variety of physiological and pathological stimuli, and thus play important roles in cell cycle control, development, metabolism, pathogenesis, reproduction, response to intercellular signals, and environment. FOXP3 is a member of the transcription factor family of FOX proteins and has major functions in autoimmune homeostasis and cancer development. Herein, we show for the first time that FOXP3 acts as a transcriptional activator of the human *UBC9* gene in MCF7 breast cancer cells.

Hundreds of FOXP3 target genes have been identified in both Treg cells and cancer cells so far, including *HER2/ERBB2* [11], *SKP2* [14], *CD44* [13], *BRCA1* [12], *p21* [32], and *LATS2* [33]. Importantly, our data showed that UBC9 (both mRNA and proteins) are up-regulated by FOXP3 in human MCF7 breast cancer cells. Indeed, we observed that the induction of FOXP3 by the removal of doxycycline from FOXP3-Tet-off MCF7 cells enhances UBC9 expression (but decreases SKP2 expression, which is consistent with the previous finding [14]) in a time-dependent manner. Our promoter analysis further supports that FOXP3 mainly binds to a FOX response element in the proximal promoter region to regulate the human *UBC9* gene. Our data suggest that *UBC9* is the novel target gene for FOXP3.

Post-translational modifications (such as phosphorylation, ubiquitination, acetylation, and SUMOylation), which regulate a large percentage of all protein functions in cells, is the core principle in biochemistry and cell biology. As a transcription factor, FOXP3 has been shown to be phosphorylated at S418 in 2008. In the present work, we demonstrate that the loss of phosphorylation on FOXP3 S418 (S418A) only mildly reduces its activity in regulating the *UBC9* promoter (Figure 5). This observation is consistent with the previous report using *IL2* promoter [29], suggesting that the additional phosphorylation site(s) might be present and important for FOXP3. In 2013, the phosphorylation of FOXP3 at Y342 by LCK was discovered [30]. In the present study, we show that the loss of phosphorylation on FOXP3 Y342 (Y342F) significantly reduces its activity in regulating the *UBC9* promoter (Figure 5). This further highlights that Y342, located in the forkhead DNA binding domain, is a critical phosphorylation site for FOXP3. Ubiquitination and acetylation (another two major post-translational modifications) are complex homeostatic processes that regulate the majority of transcription factors. FOXP3 has been shown to be targeted by acetylation and ubiquitination systems at various sites, including K31 (located in N-terminal repressor domain), K263, and K268 (both K263 and K268 are located in leucine zipper domain) [31]. In the present study, we indicate that the loss of acetylation and/or ubiquitination on FOXP3 K263 and K268 (K263R and K263RK268R) significantly reduces its activity in regulating the *UBC9* promoter (Figure 5). However, the de-acetylation of FOXP3 at K31 (K31R) does not affect the FOXP3-mediated *UBC9* promoter activity. These results suggest that the sites of post-translational modifications in forkhead DNA binding and leucine zipper domains are more important for FOXP3-mediated *UBC9* promoter activities. Another post-translational modification, SUMOylation, has emerged as an important process in a variety of biological events and regulations, to environmental cues. Previous studies, including ours, have shown that both FOXP1 and FOXP2 are the substrates of SUMOylation [28,34]. However, we have not found SUMOylation site(s) for FOXP3 based on our in vitro and in vivo studies (data not shown). Although FOXP3 is not the direct target of SUMOylation, our current study indicates that FOXP3 may up-regulate global SUMOylation by targeting the *UBC9* gene. Overall, our results provide further evidence that post-translational modifications are important for FOXP3 activity on its downstream target gene regulation. As FOXP3 has significant functions in immunological processes and cancer development, more studies are indeed required to further validate the role of post-translational modifications on FOXP3 functions in vivo.

In summary, we demonstrate that FOXP3, through FOX response elements, activates *UBC9* promoter activity and post-translational modifications (phosphorylation, acetylation, and ubiquitination) may regulate the FOXP3 activity. Collectively, our results not only extend the conclusion that FOXP3 is involved in the SUMOylation process, but also provide the consistent evidence that post-translational modifications play a role in regulating FOXP3 activity.

## 4. Materials and Methods

### 4.1. Reagents and Chemicals

All of the cell culture reagents were purchased from Thermo Fisher Scientific (Waltham, MA, USA). Antibodies against FOXP3, SKP2, UBC9, and β-Actin were purchased from Santa Cruz Biotechnology Inc. (Santa Cruz, CA, USA). The luciferase activity was measured using the Dual-Luciferase Assay System (Promega, Madison, WI, USA).

### 4.2. DNA Constructs

Human *FOXP3*-pcDNA6 expression plasmid was described previously [11,15,16]. S418A, Y342F, K31R, K263R, and K263RK268R *FOXP3* expression plasmids, as well as the FOXP3 response element mutations (−310 bp mutant (GCCAACA → GCCGGCA) and −2182 bp mutant (GTTGGC → GCCGGC) *UBC9* promoter-LUC plasmids were created by PCR-based mutagenesis (QuikChange Lightning site-directed mutagenesis kit, Agilent/Strategene, La Jolla, CA, USA). The human *UBC9* promoter (−2392/+124 bp) pGL3 plasmid was kindly provided by Dr. Hamann (German Cancer Research Center, Heidelberg, Germany). The human *UBC9* promoter deletion constructs were then generated by the removal of specific fragments of DNA sequence in Yang lab. All of the constructs were verified by nucleotide sequencing.

### 4.3. Cell Culture and Transfection

MCF7 and HEK293 cells were obtained from the American Type Culture Collection (Manassas, VA, USA). The cells were cultured in DMEM supplemented with 10% fetal bovine serum and antibiotics (GIBCO/Life Technologies, Grand Island, NY, USA) in humidified air (5% CO_2_ at 37 °C), and were cultured for less than six months. FOXP3-Tet-off MCF7 cells were previously established and maintained in 1 μg/mL doxycycline (Dox), as described previously [14,32]. All of the cell lines were tested Mycoplasma-free, as determined by a LookOut Mycoplasma PCR Detection Kit (Sigma, St. Louis, MO, USA). The transfections were performed using Fugene HD Transfection Reagent (Roche, Madison, WI, USA) in MCF7 and HEK293 cells, according to manufacturer’s instructions.

### 4.4. UBC9 Luciferase Reporter Assay

Dual-luciferase reporter assays were performed according to the manufacturer’s instructions. The cells were cultured in 24-well plates overnight and then transiently transfected with *UBC9* promoter-firefly luciferase plasmid and internal control pRL-TK plasmid (which encodes Renilla activity). The cells were harvested and lysed in passive lysis buffer, 48 h after transfection. The firefly luciferase activity was measured and normalized with the Renilla activity. Data were presented as relative LUC activity, where the values of the control groups were set to 1 for each cell line. All of the experiments using the transfection method were performed three times in triplicate setting.

### 4.5. RT-PCR and Real-Time ChIP

The total RNA from the MCF7 cells was extracted using the TRIzol reagent, and treated with DNase (Ambion, Austin, TX, USA) to remove the genomic DNA. The RNA concentration was quantified by ultraviolet spectrometry. One microgram of total RNA was converted into cDNA, using the iScript kit (Bio-Rad, Hercules, CA, USA), according to the manufacturer’s instructions. The final cDNA product was purified and eluted in a Tris-EDTA buffer, using QIAquick PCR purification kits (QIAGEN, Germantown, MD, USA), according to the manufacturer’s instructions. Quantitative PCR (using 1–10 ng cDNA per μL) was performed on an ABI 7500 sequence detector (Applied Biosystems, Foster City, CA, USA), using TaqMan Universal PCR Master Mix Kit (Applied Biosystems, Foster City, CA, USA), according to the manufacturer’s instructions. Two primers (5′-GAT GAT TAT CCA TCT TCG CCA C-3′ and 5′-GTC GCT GCT TAT GAG GGC G-3′) were used to amplify the 289-bp human *UBC9* fragments. Two primers (5′-CAT CAC CAT CTT CCA GGA GCG AG-3′ and 5′-GTC TTC TGG GTG GCA GTG ATG G-3′) were used to amplify the 341 bp human glyceraldehydes-3-phosphate dehydrogenase (*GAPDH*) fragments. For a real-time ChIP analysis, the extracted DNA fragments were quantified by real-time PCR, using pairs of primers that covered the FOXP3 response region within the human *UBC9* promoter. The primers used for the −310 RE PCR were CTTTGGGAGGCCGAGG (forward) and ACCACCACACCCGGC (reverse). The primers used for the −2182 RE PCR were GTCGGCGAAATGC (forward) and CAGGACCGGGTCG (reverse).

### 4.6. Western Blot Analysis

MCF7 cells (2 × 10^6^) were seeded onto 100 mm cell culture dishes or (5 × 10^5^) were seeded onto 6-well cell culture plates overnight, before the transient transfection procedure. The cells were harvested 48 h after transient transfection and after centrifuge, the cell pellets were lysed in a RIPA buffer. Protein lysates were placed onto a rotator to rotate at 4 °C for 30 min, and the protein concentrations of the high-speed supernatant were quantified using the BCA™ Protein Assay kit assay (Pierce/Thermo Scientific, Rockford, IL, USA). Immunoblotting was performed, as previously described [23,27,28]. Equivalent quantities of protein (40–50 µg) were resolved on polyacrylamide-SDS gels, transferred to PVDF membrane (Bio-Rad, Hercules, CA, USA), and immunoblotted with specific antibodies. The immune detection was done with the Supersignal West Dura Extended Duration Substrate kit (Pierce Chemical Co., Rockford, IL, USA). The intensity of the protein band was quantified by the NIH ImageJ program.

### 4.7. Statistical Analysis

We performed statistical analyses for the significance of the differences between two measurements by using the unpaired 2-tailed Student’s *t*-test. When more than two groups were compared, a one-way ANOVA was performed. For each test, *p* < 0.05 was considered statistically significant between groups.

## 5. Conclusions

In summary, this investigation has demonstrated that FOXP3, one of the FOX protein family members, is a novel activator of the human *UBC9* promoter in MCF7 breast cancer cells. Moreover, the post-translational modifications (phosphorylation, acetylation, and ubiquitination) may play a critical role for FOXP3’s transcriptional activity. Our study also adds a new layer of information to the previous understanding of how FOXP3 functions in Treg development and acts as a tumor suppressor in cancer development.

## Abbreviations

FOXP3	Forkhead Box Protein P3
UBC9	SUMO-conjugating enzyme UBC9
SUMO	small ubiquitin-like modifier
SUMOylation	a post-translational modification process involving SUMO addition
BRCA1	breast cancer type 1 susceptibility protein
CD44	CD44 antigen
HER2/ERBB2	receptor tyrosine-protein kinase erbB2
SKP2	S-phase kinase-associated protein 2
MCF7	a breast cancer cell line
LATS2	serine/threonine-protein kinase LATS2
LCK	Lymphocyte-specific protein tyrosine kinase
